# Fatigue, Depression, and Anxiety Among Ambulating Multiple Sclerosis Patients

**DOI:** 10.3389/fimmu.2022.844461

**Published:** 2022-03-29

**Authors:** Safanah AlSaeed, Tamadher Aljouee, Nuha M. Alkhawajah, Rola Alarieh, Hanan AlGarni, Salman Aljarallah, Mohsen Ayyash, Amani Abu-Shaheen

**Affiliations:** ^1^Physical Therapy Department, King Fahad Medical City, Riyadh, Saudi Arabia; ^2^College of Medicine, King Saud University, Riyadh, Saudi Arabia; ^3^Department of Neurology, King Fahad Medical City, Riyadh, Saudi Arabia; ^4^School of Mathematical Sciences, Universiti Sains Malaysia, Penang, Malaysia; ^5^Research Center, King Fahad Medical City, Riyadh, Saudi Arabia

**Keywords:** multiple sclerosis, depression, fatigue, physical activity, quality of life, Riyadh, anxiety, Kingdom of Saudi Arabia

## Abstract

**Background:**

Multiple sclerosis (MS) is an inflammatory disease associated with adverse effects: including depression, anxiety, fatigue, which may affect physical activity and the quality of life (QoL) among patients with MS (pwMS).

**Objective:**

This study aims to assess the prevalence of depression, anxiety, and fatigue among pwMS who have no physical disability in Saudi Arabia, and demonstrate any correlation between these factors and physical activity as well as the QoL.

**Methods:**

A cross-sectional study was conducted in the Neuroimmunology outpatient clinics in King Fahad Medical City (KFMC) and King Saud University Medical City (KSUMC) in Riyadh City, KSA. The Arabic version of the Hospital Anxiety and Depression Scale (HADS) was used to measure anxiety and depression levels. The HADS scores were then categorized into three levels according to the total points: normal (0–7 points), borderline (7–10 points), and anxiety/depression (11 – 21 points). The Arabic version of the Fatigue Severity Scale (FSS) was used to measure fatigue (cut-off point ≥5). The physical activity was measured by the Arabic version of the short form of the International Physical Activity Questionnaire (IPAQ), which measure time spent walking, moderate- and vigorous-intensity physical activity of at least 10 minutes duration. The QoL was also measured by the Arabic version of the EuroQOL five-dimensional (EQ-5D-3L) instrument (i.e., mobility, self-care, usual activities, pain/discomfort, and anxiety/depression).

**Results:**

A total of 323 pwMS participated in this study, 83 had scores that indicated anxiety (25.7%) and 44 had depression (13.6%). The majority of patients had scores with the normal range of depression and anxiety (70% and 57% respectively). The mean of EuroQol Group visual analogue scale (EQ-VAS) score was 80.43 (SD=19.8). 156 (48.3%) out of 323 pwMS reported fatigue while the remainder had no fatigue (n=167, 51.7%). The results indicate that only 143 patients (44.3%) had participated in vigorous physical activity during the last 70 days, with a median of 3 days per week (IQR= 5–3) and a median of 60 minutes per day 0 (Interquartile range: IQR = 60–30). Only 149 patients (49.2%) had patricpated in moderate physical activities during the previous week with a median of 3 days per week (IQR = 5–3) and a median of 40 minutes per day (IQR = 60–30). 194 patients had participated in walking activities (60.0%) with a median of 5 days per week (IQR = 7–3) and a median of 45 minutes per day (IQR = 60–30). The results revealed that fatigue was positively correlated with depression (r = 0.407, p-value < 0.001) and anxiety (r = 0.289, p-value < 0.001).

**Conclusion:**

The current study shows depression, anxiety, and fatigue tend to be correlated and clustered together among pwMS in our cohort. However, fatigue is not associated with the intensity of physical activity undertaken. The results of this study are important for the improvement of the clinical management of MS patients.

## Introduction

One of the most common neurological disorders that can affect young adults is multiple sclerosis (MS) (1). It is a chronic, inflammatory, autoimmune disease that affects the central nervous system. The inflammation leads to demyelination and axonal loss, manifesting as different cognitive, motor, or sensory symptoms depending on the lesions’ location (2). The current prevalence of MS in the Kingdom of Saudi Arabia (KSA) is estimated to be around 62 patients per 100,000 Saudi nationals, although unfortunately at present there is no National Regisry of MS (3). In general, MS tends to affects females more than males, with an estimated 3:1 female to male ratio globally (2). In KSA, this ratio was estimated at approximately 2:1 woman to men ratio (3). The majority of MS patients ambulating normally and with no disability according to their median expanded disability status score (EDSS) score 1 (3). The disease’s etiology remains unclear (4), but it is thought to be a consequence of a complex interaction between genetic and environmental factors (2, 5).

Depression, anxiety, and fatigue are common among patients with MS (pwMS) and do affect their quality of life (QoL) and physical activity (6–11). In some studies, the prevalence of depressive symptoms and anxiety ranged between 14-54% and 14-41% respectively (6–8, 11). Depression and anxiety have an unpredictable disease nature and are considered to be the most disabling symptoms that affect the QoL and general health in MS patients (11, 12). Associations between depression and disability and non-motor symptoms such as fatigue have been studied but the results are inconsistent (12–15). For example, some studies reported a direct association (12, 14, 16–18) while others did not (19, 20). Furthermore, anxiety was found to be associated with chronic pain while it was moderately associated with disability and fatigue (11, 12, 14). Some studies reported that up to 90% of pwMS had symptoms of fatigue, whereby they defined fatigue as tiredness, low energy, or exhaustion, and that these symptoms might be triggered by activities or increased temperature (11, 12, 14). Factors contributing to fatigue may include the individual presentation of the disease, some treatment side effects, functional status impairment, weakness, pain, and nocturia (21). Moreover, a study by Ayache and Chalah reviewed various causes of fatigue in pwMS which included anemia, vitamin-deficiencies, endocrine disorders, sleep disorders, psychiatric comorbidities, psychological burden, and medication side effects (22). However, there were no clear causes of fatigue in pwMS have been found and the related-literature is inconclusive.

The impact of fatigue, depression and anxiety symptoms on patients QoL should not be ignored. Several studies have investigated the relationship between QoL and depression, anxiety, stress, and fatigue (23–25). The majority of these studies indicated that these factors were significantly correlated with the QoL (23–26). A recent study in KSA found that the majority of Saudi pwMS reported that overthinking about social life problems, mood swings, and sleep disturbance had an impact specifically on disease relapse and its severity (27). Furthermore, the emotional burden, mood swings, and difficulty in making decisions can also have an impact on the disease course, particularly in terms of relapse or its severity as well as in the QoL and psychological wellbeing (27).

Therefore, focusing on improving and alleviating these adverse MS-related symptoms is crucial. However, the prevalence of depression, anxiety, and fatigue in pwMS is not thoroughly studied in Saudi Arabia. Thus, the purpose of this study is to identify the prevalence of depression, anxiety, and fatigue among ambulating pwMS in Saudi Arabia. Furthermore, this study intends to examine the correlations between QoL, physical activity and these symptoms.

## Methods

### Study Design and Settings

This is a cross-sectional study, conducted in the Neuroimmunology outpatient clinics in King Fahad Medical City (KFMC) and King Saud University Medical City (KSUMC) in Riyadh, KSA. Patients were also recruited from a database provided by the ARFA MS association. ARFA association is a non-profit organization helping pwMS approved by the Saudi Ministry of Human Resources and Social Development. The ARFA MS association accepts MS patients with medical report to confirm the diagnosis.

### Study Participants

The study population consisted of patients diagnosed with MS based on the McDonald Criteria (2017) by a neurologist attending KFMC or KSUMC neurology clinics. All patients were aged 18 years and above, with at least a 1-year history of MS with no walking difficulty based on the EQ-5D-3L questionnaire, had no history of relapse in the previous eight weeks, were deemed eligible to participate in this study. Those patients with difficulty in walking and/or those who had a relapse within the previous 8 weeks were excluded from this study since they will probably report high fatigue levels to avoid bais. As well as patients who are illiterate or non-arabic speakers were excluded.

### Estimated Sample Size

A total number of 2,313 patients have enrolled in the National MS Registry (NMSR), which is approximately 38% of the estimated number of patients with MS in KSA. More than a half of the patients (80%) have no or minimal disability (3). Hence the expected population of 1700 patients fits the inclusion criteria. While presuming 10% of the expected population are post exclusion of illiterate (N=1530), and assuming the response rate is around 30% based on the clinical experience and as discussed with the research ethics committee; the estimated sample size is N=323, as derived with the help of Cochran’s formula outlined below.

### Data Collection Methods

The data was collected using a self-reported questionnaire, which was available in the Arabic language. The questionnaire contains five sections. The first section the demographic data (age, gender, marital status, education, area of residence, and current work status) and clinical details (number of years since diagnosis and date of last MS relapse) The remaining sections measure the outcomes of this study. These outcome measures are aim to ascertain many life domains such as physical activity, depression, anxiety, fatigue, disability, and QoL. These outcomes are provided in more detail below.

Hospital Anxiety and Depression Scale (HADS) (28) includes 14 items assessing anxiety (7−item) and depression (7−item), which are rated from 0 to 3. The scores in each subscale are computed by summing the corresponding items, with maximum scores of 21 for each subscale. A score of 0–7 is considered normal, 8–10 as a borderline case, and 11–21 as a case (of anxiety or depression) (28). The Arabic version of HADS is a reliable and valid tool to use with pwMS. A systematic translation process was used to translate the original English HADS into Arabic and validated after a pilot study; reliability was tested by using internal consistency examination (29).

Physical activity was measured by the short version of the International Physical Activity Questionnaire (IPAQ) (30). Which consists of 7 questions that measure the intensity of walking, moderate-intensity activities, and vigorous-intensity through the previous week and the usual occurrence. Computation of the total score for the short form requires the summation of the duration (in minutes) and frequency (days) of walking, moderate-intensity, and vigorous-intensity activities. MET level x minutes of activity x events per week for each of walking, moderate- and vigorous-intensity activities were calculated as follows: walking = (3.3 × walking minutes × number of walking days); moderate activity = (4.0 × moderate activity minutes × moderate activity days); vigorous activity = (8.0 × vigorous activity minutes × vigorous activity days). There are three categories of physical activity used to classify participants (low, moderate, and high) according to the scoring system provided by IPAQ (www.ipaq.ki.se) (31). The Arabic short self-report IPAQ form was validated and the reliability confirmed. It was translated and adapted to the Arabic language and then subjected to back-translation (31).

The EuroQOL five-dimension questionnaire EQ-5D-3L is widely-used globally and has been translated into approximately 150 of languages (32). It is a short patient-reported outcome measure that consists of two sections. The first section measures five dimensions (mobility, self-care, usual activities, pain/discomfort, and anxiety/depression) with three response levels per dimension – ‘no problems’, ‘some problems’, or ‘extreme problems’. pwMS in our cohort were asked to provide information regarding their health status by checking the box that indicated the most appropriate statement in every item of each dimension. The result of each dimension is given a 1-score number that exhibits the selected level for that dimension. The scores of all dimensions are then combined and form a 5-scores number that describes the patient’s health state. The second section is a visual analogue scale (VAS) rated from 0 (worst health imaginable) to 100 (best health imaginable), which gave an overall impression of the patients’ current wellbeing (32). We used previously validated Arabic version of EQ-5D-3L for evaluating health-related QoL in KSA (33).

Fatigue is measured by the Fatigue Severity Scale (FSS), which was found to be sensitive, reliable, and consistent in pwMS with a good response rate of 0 (34). It is a subjective measure of fatigue that is based on the self-reported assessment by pwMS (22). The FSS consists of nine statements describing the severity and impact of fatigue, with possible responses ranging from 1 (strongly disagree) to 7 (strongly agree). Total FSS scores are usually reported as the mean score of the nine items, with higher scores indicating increased severity (34). A cut-off point of ≥ 5 points was considered as the presence of fatigue. The Arabic version of the FSS demonstrated acceptable test-retest reliability, internal consistency, and psychometric properties and was able to differentiate between pwMS and has been shown capable of differentiating between healthy subjects (35).

The questionnaire of the current study was available online *via* a Google Forms link which was distributed to the patients, the respondents were asked to self-report the questionnaire within 24 hours of receiving the link. The questionnaire link was distributed to the participants through social media platforms such as WhatsApp, Twitter, and Emails.

### Statistical Analysis

Demographic and clinical characteristics of the study participants are reported as mean (standard deviation; SD) or median (Interquartile range; IQR) for continuous variables as appropriate. Additionally, categorical variables were reported as counts and percentages or in bar charts as appropriate. Chi-square tests of association were performed to examine the association between two categorical variables. Additionally, the normality of data distribution was examined by the Kolmogorov-Smirnov test. Differences in mean or median scores of each scale were examined using non-parametric tests because the distribution of outcome variables was not normally distributed. Specifically, the Mann-Whitney test was used to assess the differences in outcomes between two independent samples, and the Kruskal Wallis test was used to test for differences in the mean scores of each scale because in involves more than two independent samples. For Kruskal Wallis test with the statistically significant results, the Dunn procedure was used, which accounts for type I error and thus reduces the likelihood of false positive-results (36). The correlation between the continuous scores were assessed by calculating Spearman’s correlation coefficient. All statistical analyses were performed using SPSS 24.0 software (SPSS Inc., Chicago, IL, USA) package; two-tailed test and a p-value of less than 0.05 was considered significant.

### Ethical considerations

An electronic informed consent was obtained before filling out the questionnaires. All data has been kept confidential and has only been analyzed after the subjects‘ approval without any personal identifiers.

## Results

More than 2000 patients received the questionnaire and 616 patients were answered it. Only 323 pwMS of these met the study inclusion criteria. The majority of participants were females (n=277, 70.3%), Saudis (n=293, 90.3%), and resided in the central region of KSA (n=213, 65.9%). One hundred and forty-five patients (44.9%) belonged to the 18-29 years age group. More than one-half of the respondents were married (n=171, 52.9%) and had university education (n=193, 59.3%). Approximately one-half of the participants were currently employed (n=159, 49.2%). The average length of time senice diagnosis was 5.5 (*SD* ± 4.7) years, while the average number of years since the last MS relapse was 2.46 (*SD* ± 2.3) (**Table 1**).

**Table 1:** Descriptive statistics of the study sample.

	N	%
**Age Groups**		
18 -29	145	44.9
30 -39	132	40.9
40 – 49	40	12.4
≥50	6	1.9
**Gender**		
Male	96	29.7
Female	227	70.3
**Marital status**		
Married	171	52.9
Single	152	47.1
**Region**		
Central	213	65.9
Eastern	34	10.5
Western	54	16.7
Northern	18	5.6
Southern	4	1.2
**Nationality**		
Saudi	293	90.3
Non-Saudi	30	9.7
**Education**		
Secondary or lower	53	16.4
Diploma	35	10.8
University	193	59.8
Postgraduate	42	13.0
**Work Status**		
Employed	159	49.2
Unemployed	63	19.5
Student	47	14.6
Homemaker	54	16.7
	**Mean**	**SD**
**No. of years after diagnosis**	5.5	4.7
**Last MS relapse**	2.46	2.3

### Depression and Anxiety Prevalence

**Figure 1:**
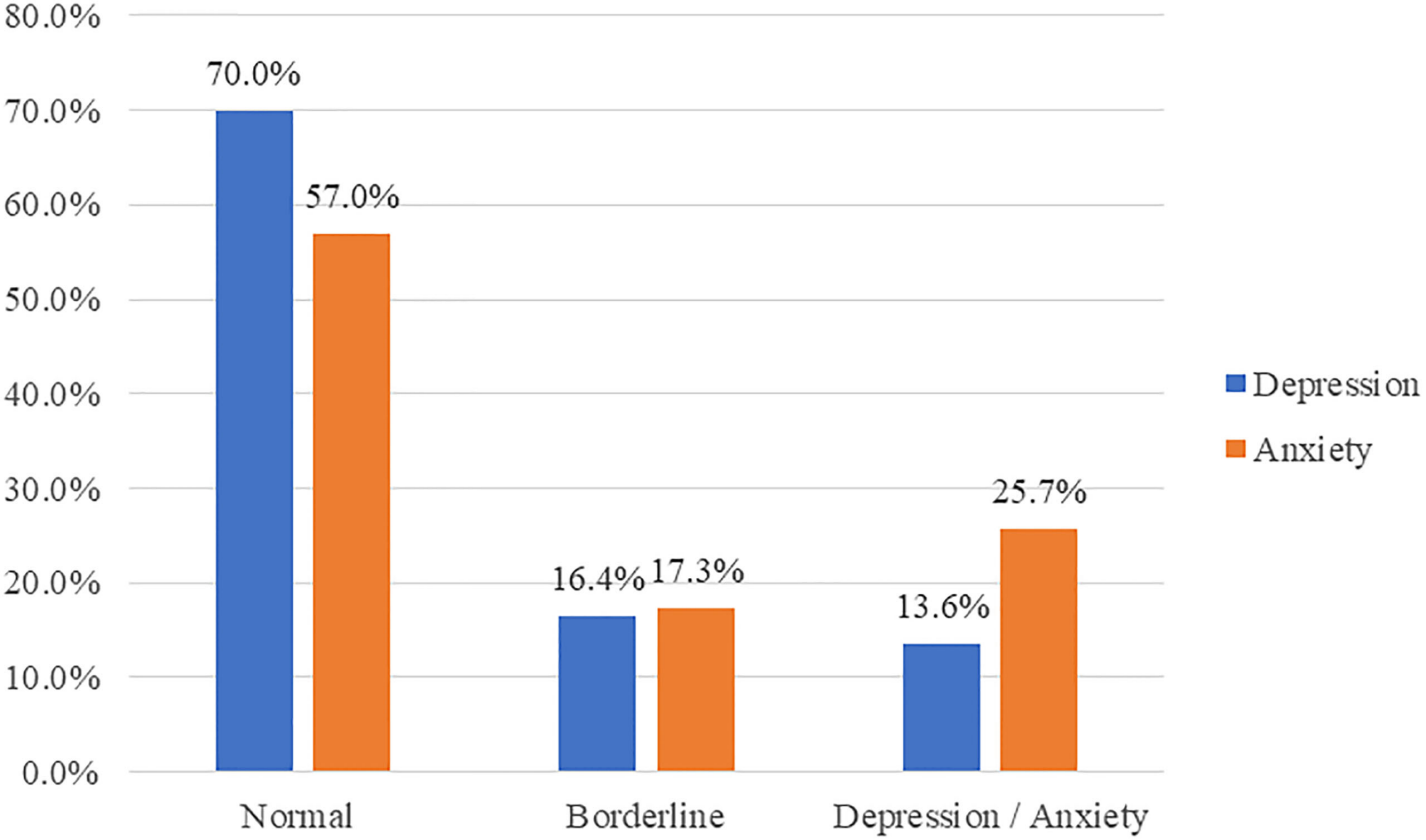
Prevalence of anxiety and depression using HADS.

**Figure 1** shows the prevalence of anxiety and depression among our cohort as measured by the HADS outcome. The majority of participants had normal depression and anxiety levels. However, more patients had anxiety than depression (25.7% vs. 13.6% respectively).

In this study, the participants exhibited a mean score of depression of 5.63 (SD=4.2) and a mean score for anxiety of 7.34 (SD=4.9), as indicated in **Table 2**. Statistical differences in the mean scores of depression and anxiety by patients’ characteristics are also shown in **Table 2**. The mean scores of depression and anxiety did not differ significantly with gender, marital status, nationality, educational level, or work status (*P*_value *>*0.05). However, they were statistically significant differences for anxiety by age groups (*P*_value = 0.011) but not for depression (*P*-value = 0.608). Specifically, patients aged 18 – 29 years or 50 years and above had a higher mean score of anxiety than those aged 30 – 39 years (*P*_value = 0.005 and 0.043 respectively). Also, statistically significant differences in mean scores of depression and anxiety were evident among pwMS by region of residence (*P*_value < 0.05). Patients who lived in the central and western regions had significantly higher depression and anxiety scores than those who lived in the eastern region (*P*_value < 0.05). The duration in time snice diagnosis of MS and the number of years since the last MS relapse were significantly and negatively correlated with anxiety but not with depression.

**Table 2:** Depression and anxiety total mean scores and by demographic characteristics.

	Depression	*P*_value	Anxiety	*P*-value
**Total score**	5.63 ± 4.2		7.34 ± 4.9	
***Gender***				
Male	5.59 ± 4.2	0.988	7.31 ± 4.8	0.925
Female	5.64 ± 4.3		7.42 ± 4.9	
***Age Groups***				
18 – 29	5.83 ± 4.5	0.608	8.09 ± 4.8	0.011^a^
30 – 39	5.38 ± 3.9		6.57 ± 4.6	
40 – 49	5.45 ± 4.4		7.05 ± 5.6	
≥50	7.50 ± 4.8		10.83 ± 5.0	
***Marital Status***				
Married	5.66 ± 4.3	0.968	7.67 ± 5.0	0.379
Single	5.59 ± 4.2		7.08 ± 4.8	
*Region*				
Central	5.93 ± 4.4	0.036^b^	7.44 ± 4.9	0.032^c^
Eastern	3.85 ± 3.6		5.44 ± 4.9	
Western	6.07 ± 4.5		8.5 ± 4.9	
Northern	4.44 ± 2.7		7.22 ± 4.1	
Southern	3.75 ± 2.5		7.25 ± 7.5	
***Nationality***				
Saudi	5.52 ± 4.2	0.302	7.24 ± 4.9	0.069
Non-Saudi	6.60 ± 4.9		8.87 ± 4.9	
***Education***				
Secondary or lower	6.81 ± 4.5	0.052	7.49 ± 5.2	0.846
Diploma	4.48 ± 3.2		7.49 ± 4.1	
University	5.39 ± 4.3		7.25 ± 4.9	
Postgraduate	6.17 ± 4.3		7.81 ± 5.1	
***Work status***		0.072		0.069
Employed	5.28 ± 4.0		7.08 ± 4.8	
Unemployed	5.21 ± 4.0		6.71 ± 5.1	
Student	5.29 ± 4.5		8.00 ± 5.2	
Housewife	5.38 ± 4.4		7.29 ± 4.7	
**No. of years since diagnosis**	- 0.071^d^	0.206	- 0.112^d^	0.045
**Last MS relapse **	- 0.058^d^	0.342	- 0.166^d^	0.007

a: P = 0.005 for 18 – 29 versus 30 – 39; 0.113 for 18 -29 versus 40 – 49; 0.140 for 18 – 29 versus ≥ 50; 0.872 for 30 – 39 versus 40 – 49; 0.043 for 30 – 39 versus ≥ 50; 0.160 for 40 – 49 versus ≥ 50.

b: P = 0.004 for central versus eastern; 0.850 for central versus western; 0.273 for central versus northern; 0.341 for central versus southern; 0.010 for eastern versus western; 0.247 for eastern versus northern; 0.801 for eastern versus southern; 0.286 for western versus northern; 0.363 for western versus southern; 0.594 for northern versus southern.

c: P = 0.009 for central versus eastern; 0.155 for central versus west; 0.962 for central versus northern; 0.678 for central versus southern; 0.001 for eastern versus western; 0.073 for eastern versus northern; 0.697 for eastern versus southern; 0.382 for western versus northern; 0.504 for western versus southern; 0.652 for northern versus southern.

d: Correlation coefficient.

### EQ-5D-3L

**Figure 2:**
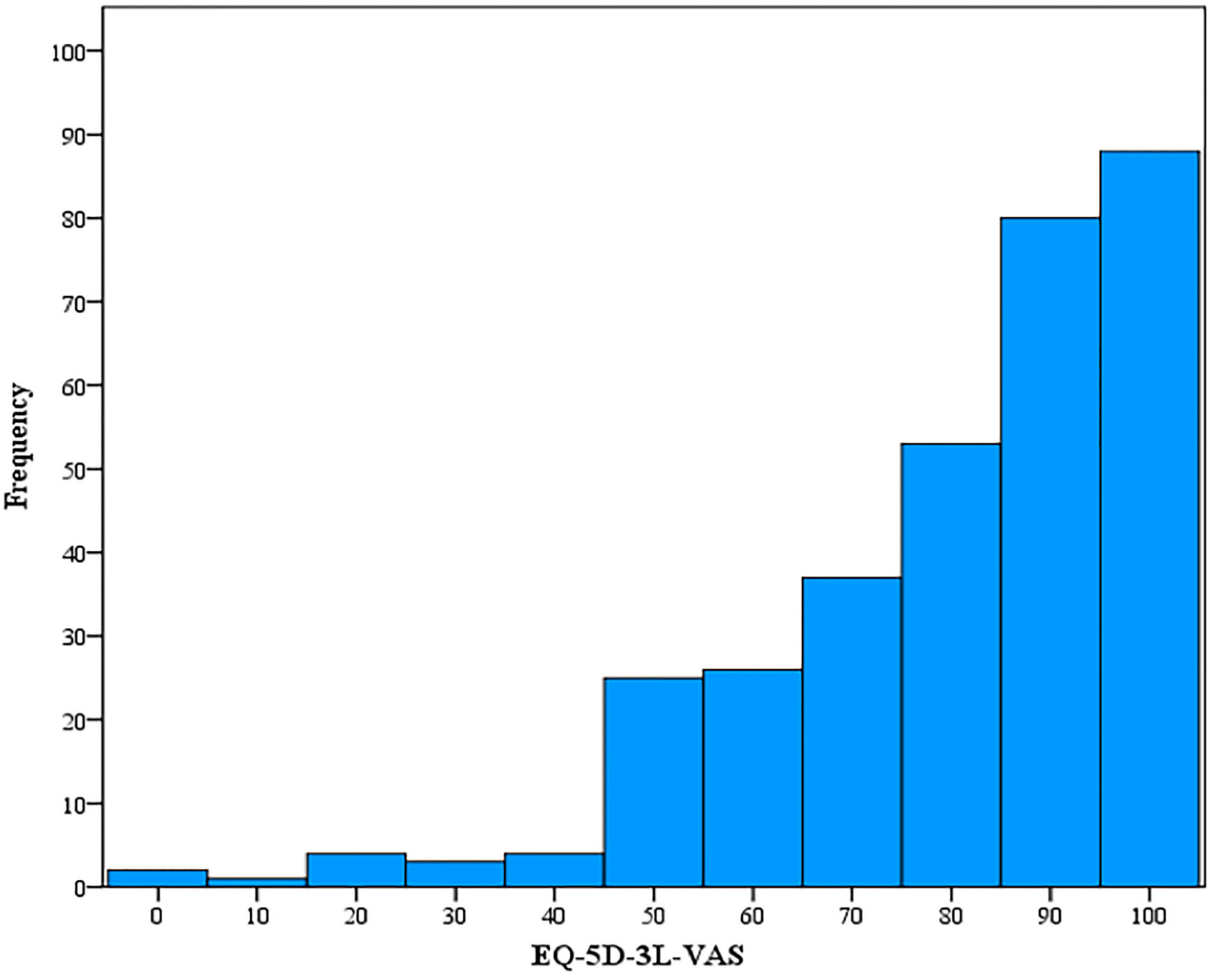
EQ-5D-3L VAS Frequency Distribution.

According to EQ-5D-3L, a Full Health State was reported in eighty-one participants (25.1%) (i.e., 11111), while no participants exhibited the worst health state (i.e., 33333). No respondents had issues with mobility (n = 323, 100%). the vast majority of the participants had no problems in self-care (n = 313, 96.9%), or usual activities (n = 241, 74.6%). On the other hand, more than one-half had reported having pain or discomfort (n = 171, 52.9%). While half of the patients (n = 163) had no reported problems with anxiety or depression (**Table 3**). Regarding the patients’ self-assessment of their health, the mean EuroQol Group visual analogue scale (EQ-VAS) score was 80.43 (SD = 19.8). **Figure 2** shows the frequency distribution of EQ-5D-3L VAS, which indicated that most participants reported a healthy state. About 27.2% (n = 88) of patients had a full score of health status, while only two patients (0.6%) exhibited the worst health status.

**Table 3:** Frequency Distribution of EQ-5D 3L of the Total Sample and by patients' Characteristics; N (%).

	Mobility Levels			Self-care Levels			Usual activities Levels			Pain Levels			Depression/Anxiety Levels		
	no problems	some problems	extreme problems	no problems	some problems	extreme problems	no problems	some problems	extreme problems	no problems	some problems	extreme problems	no problems	some problems	extreme problems
***Total sample***	323 (100)	0 (0.0)	0 (0.0)	313 (96.9)	8 (2.5)	2 (0.6)	241 (74.6)	77 (23.8)	5 (1.5)	132 (40.9)	171 (52.9)	20 (6.2)	163 (50.5)	140 (43.3)	20 (6.2)
***Gender***															
Male	96 (100)	0 (0.0)	0 (0.0)	89 (92.7)	5 (5.2)	2 (2.1)	76 (79.2)	17 (17.7)	3 (3.1)	39 (40.6)	50 (52.1)	7 (7.3)	56 (58.3)	34 (35.4)	6 (6.3)
Female	257 (100)	0 (0.0)	0 (0.0)	224 (98.7)	3 (1.3)	0 (0.0)	165 (72.7)	60 (26.4)	2 (0.9)	93 (41.0)	121 (53.3)	13 (5.7)	107 (47.1)	106 (46.7)	14 (6.2)
***Age Groups***															
18 – 29	145 (100)	0 (0.0)	0 (0.0)	142 (97.9)	1 (0.7)	2 (1.4)	107 (73.8)	36 (24.8)	2 (1.4)	55 (37.9)	82 (56.6)	8 (5.5)	62 (42.8)	72 (49.7)	11 (7.6)
30 – 39	132 (100)	0 (0.0)	0 (0.0)	128 (97.0)	4 (3.0)	0 (0.0)	107 (81.1)	24 (18.2)	1 (0.8)	60 (45.5)	65 (49.2)	7 (5.3)	77 (58.3)	49 (37.1)	6 (4.5)
40 – 49	40 (100)	0 (0.0)	0 (0.0)	38 (95.0)	2 (5.0)	0 (0.0)	26 (65.0)	14 (35.0)	0 (0.0)	15 (37.5)	22 (55.0)	3 (7.5)	22 (55.0)	16 (40.0)	2 (5.0)
50	6 (100)	0 (0.0)	0 (0.0)	5 (83.3)	1 (16.7)	0 (0.0)	1 (16.7)	3 (50.0)	2 (33.3)	2 (33.3)	2 (33.3)	2 (33.3)	2 (33.3)	3 (50.0)	1 (16.7)
***Marital status***															
Married	171 (100)	0 (0.0)	0 (0.0)	166 (97.1)	3 (1.8)	2 (1.2)	129 (75.4)	39 (22.8)	3 (1.8)	70 (40.9)	90 (52.6)	11 (6.4)	79 (46.2)	77 (45.0)	15 (8.8)
Single	152 (100)	0 (0.0)	0 (0.0)	147 (96.7)	5 (3.3)	0 (0.0)	112 (73.7)	38 (25.0)	2 (1.3)	62 (40.8)	81 (53.3)	9 (5.9)	84 (55.3)	63 (41.4)	5 (3.3)
***Region***															
Central	213 (100)	0 (0.0)	0 (0.0)	208 (97.7)	3 (1.4)	2 (0.9)	156 (73.2)	54 (25.4)	3 (1.4)	100 (46.9)	107 (50.2)	6 (2.8)	108 (50.7)	92 (43.2)	13 (6.1)
Eastern	34 (100)	0 (0.0)	0 (0.0)	33 (97.1)	1 (2.9)	0 (0.0)	27 (79.4)	7 (20.6)	0 (0.0)	12 (35.3)	20 (58.8)	2 (5.9)	26 (76.5)	7 (20.6)	1 (2.9)
Western	54 (100)	0 (0.0)	0 (0.0)	51 (94.4)	3 (5.6)	0 (0.0)	40 (74.1)	12 (15.6)	2 (3.7)	16 (29.6)	28 (51.9)	10 (18.5)	20 (37.0)	29 (53.7)	5 (9.3)
Northern	18 (100)	0 (0.0)	0 (0.0)	17 (94.4)	1 (5.6)	0 (0.0)	15 (83.3)	3 (16.7)	0 (0.0)	3 (16.7)	14 (77.8)	1 (25.0)	7 (38.9)	11 (61.1)	0 (0.0)
Southern	4 (100)	0 (0.0)	0 (0.0)	4 (100.0)	0 (0.0)	0 (0.0)	3 (75.0)	1 (25.0)	0 (0.0)	1 (25.0)	2 (50.0)	1 (25.0)	2 (50.0)	1 (25.0)	1 (25.0)
***Nationality***															
Saudi	293 (100)	0 (0.0)	0 (0.0)	286 (97.6)	5 (1.7)	2 (0.7)	214 (73.0)	75 (25.6)	4 (1.4)	121 (41.3)	155 (52.9)	17 (5.8)	155 (52.9)	119 (40.6)	19 (6.5)
Non-Saudi	30 (100)	0 (0.0)	0 (0.0)	27 (90.0)	3 (10.0)	0 (0.0)	27 (90.0)	2 (6.7)	1 (3.3)	11 (36.7)	16 (53.3)	3 (10.0)	8 (26.7)	21 (70.0)	1 (3.3)
***Education***															
Secondary or lower	53 (100)	0 (0.0)	0 (0.0)	52 (98.1)	1 (2.9)	0 (0.0)	40 (75.5)	13 (24.5)	0 (0.0)	22 (41.5)	28 (52.8)	3 (5.7)	23 (43.4)	25 (47.2)	5 (9.4)
Diploma	35 (100)	0 (0.0)	0 (0.0)	33 (94.3)	2 (5.7)	0 (0.0)	20 (57.1)	14 (40.0)	1 (2.9)	13 (37.1)	18 (51.4)	4 (11.4)	21 (60.0)	13 (37.1)	1 (2.9)
University	193 (100)	0 (0.0)	0 (0.0)	189 (98.0)	2 (1.0)	2 (1.0)	151 (78.2)	38 (19.7)	4 (2.1)	79 (40.9)	104 (53.9)	10 (5.2)	102 (52.8)	81 (42.0)	10 (5.2)
Postgraduate	42 (100)	0 (0.0)	0 (0.0)	39 (92.9)	3 (7.1)	0 (0.0)	30 (71.4)	12 (28.6)	0 (0.0)	18 (42.9)	21 (50.0)	3 (7.1)	17 (40.5)	21 (50.0)	4 (9.5)
***Work Status***															
Employed	159 (100)	0 (0.0)	0 (0.0)	154 (96.9)	5 (3.1)	0 (0.0)	126 (79.2)	32 (20.1)	1 (0.6)	69 (43.4)	81 (50.9)	9 (5.7)	88 (55.3)	64 (40.3)	7 (4.4)
Unemployed	63 (100)	0 (0.0)	0 (0.0)	62 (98.4)	1 (1.6)	0 (0.0)	51 (81.0)	12 (19.0)	0 (0.0)	29 (46.0)	31 (49.2)	3 (4.8)	35 (55.6)	24 (38.1)	4 (6.3)
Student	47 (100)	0 (0.0)	0 (0.0)	46 (97.9)	1 (2.1)	0 (0.0)	33 (70.2)	14 (29.8)	0 (0.0)	18 (38.3)	26 (55.3)	3 (6.4)	17 (36.2)	29 (61.7)	1 (2.1)
Homemaker	54 (100)	0 (0.0)	0 (0.0)	51 (94.4)	1 (1.9)	2 (3.7)	31 (57.4)	19 (35.2)	4 (7.4)	16 (29.6)	33 (61.1)	5 (9.3)	23 (42.6)	23 (42.6)	8 (14.8)

Furthermore, the this study the results as shown in **Table 3** indicate that most patients exhibited no problems in mobility, self-care, and usual activities. However, most patients had some problems in pain or discomfort levels. A total of fifty-six men (58.3%) and 107 women (47.1%) had no problems in depression or anxiety. Meanwhile, more than one-half of patients aged 18 – 29 and 50 years and above had problems with depression or anxiety. More than half of married patients, unlike single patients, had problems with anxiety and depression. Most respondents from central (n = 108, 50.7%), eastern (n = 26, 76.5%), and southern (n = 2, 50%) regions had no problems with depression or anxiety while most respondents from western (n = 26, 53.7%) and northern (n = 11, 61.1%) regions had some problems with anxiety or depression.

### Fatigue Prevalence

The results indicate that 156 (48.3%) out of 323 pwMS reported fatigue as measured by FSS. This study also stratifies fatigue prevalence by patients’ groups as indicated in **Table 4**. Of those patients with fatigue, the majority were women (n = 117, 75.0%), aged 18 – 29 years (n = 69, 44.2%), married (n = 81, 51.9%), Saudis (n = 138, 88.5%), from the central region (n = 106, 67.9%), university educated (n = 89, 57.1%), employed (n = 71, 45.5%), with depression (n = 31, 19.9%), and anxiety (n = 56, 35.9%). The results also indicate that there were no statistically significant associations between FSS and all patients’ characteristics except for depression and anxiety (**Table 4**). Furthermore, disease duration in years was not significantly associated with FSS scores (r = - 0.044, p-value > 0.05) (**Table 6**).

**Table 4:** Fatigue in multiple sclerosis patients of the total sample and by demographic characteristics, depression, and anxiety.

	Fatigue	No fatigue	*P*-value
**Total sample**	156 (48.3)	167 (51.7)	
***Gender***			
Male	39 (25.0)	57 (34.1)	0.073
Female	117 (75.0)	110 (65.9)	
***Age Groups***			
18 – 29	69 (44.2)	76 (45.5)	0.786
30 – 39	65 (41.7)	67 (40.1)	
40 – 49	18 (11.5)	22 (13.2)	
≥50	4 (2.6)	2 (1.2)	
***Marital Status***			
Married	81 (51.9)	90 (53.9)	0.724
Single	75 (48.1)	77 (46.1)	
***Region***			
Central	106 (67.9)	107 (64.1)	0.695
Eastern	9 (5.8)	25 (15.0)	
Western	28 (17.9)	26 (15.6)	
Northern	11 (7.1)	7 (4.2)	
Southern	2 (1.3)	2 (1.2)	
***Nationality***			
Saudi	138 (88.5)	155 (92.8)	0.179
Non-Saudi	18 (11.5)	12 (7.2)	
***Education***			
Secondary or lower	26 (16.7)	27 (16.2)	0.637
Diploma	17 (10.9)	18 (10.8)	
University	89 (57.1)	104 (62.3)	
Postgraduate	24 (48.3)	18 (51.7)	
***Work Status***			
Employed	71 (45.5)	88 (52.7)	0.485
Unemployed	30 (19.2)	33 (19.8)	
Student	25 (16.0)	22 (13.2)	
Homemaker	30 (19.2)	24 (14.4)	
***Depression (HADS)***			
Normal	89 (57.1)	137 (82.0)	< 0.001
Borderline	36 (23.1)	17 (10.2)	
Depression	31 (19.9)	13 (7.8)	
***Anxiety (HADS)***			
Normal	73 (46.8)	111 (66.5)	< 0.001
Borderline	27 (17.3)	29 (17.4)	
Anxiety	56 (35.9)	27 (16.2)	

### Physical Activity Using IPAQ

The descriptive statistics for the five measures of physical activity for the patients with MS are shown in **Table 5**. The results indicate that 143 patients (44.3%) had vigorous physical activities during the previous seven days with median days per week of 3 (IQR = 5 – 3) and median minutes per day of 60 (IQR = 60 – 30). Also, the result found that 159 patients (49.2%) had undertaken moderate physical activities during the previous week with median days per week of 3 (IQR = 5 – 3) and median daily minutes of 40 (IQR = 60 – 30). More than half of participants; 194 patients had undertaken walking activities (60.0%) with median days per week of 5 (IQR = 7 – 3) and median minutes per day of 45 (IQR = 60 – 30). The majority of respondents had answered do not know or not sure about how many minutes they had spent sitting on weekdays (n = 145, 70.7%). However, for those that responded the median minutes per day of sitting on a weekday was reported as 180 (IQR = 360 – 180).

**Table 5:** Descriptive statistics of physical activity measured by IPAQ.

	N (%)	Median (IQR)
**Vigorous physical Activities**		
Days per week	143 (44.3)	3 (5-3)
		
Minutes per day	142 (99.3)	60 (60 – 30)
None	0 (0)	
Don't know/Not sure	1 (0.7)	
**Moderate physical Activities**		
Days per week	159 (49.2)	3 (5 – 3)
None	164 (50.8)	
minutes per day	159 (100.0)	40 (60 – 30)
Don't know/Not sure	0 (0)	
**Walk**		
Days per week	194 (60.0)	5 (7 – 3)
None	129 (40.0)	
minutes per week	191 (98.5)	45 (60 – 30)
Don't know/Not sure	3 (2.5)	
**Sitting on a weekday**	205 (63.5)	
Minutes per day	60 (29.3)	180 (360 – 120)
Don't know/Not sure	145 (70.7)	
Vigorous MET-minutes/week	–	960 (480 – 2880)
Moderate MET-minutes/week	–	600 (1200 – 240)
Walking MET-minutes/week	–	594 (1039 – 239)
Total physical activity MET-minutes/week	133 (41.2)	2838 (4802 – 1364)

Time in hours is converted to minutes.

**Figure 3:**
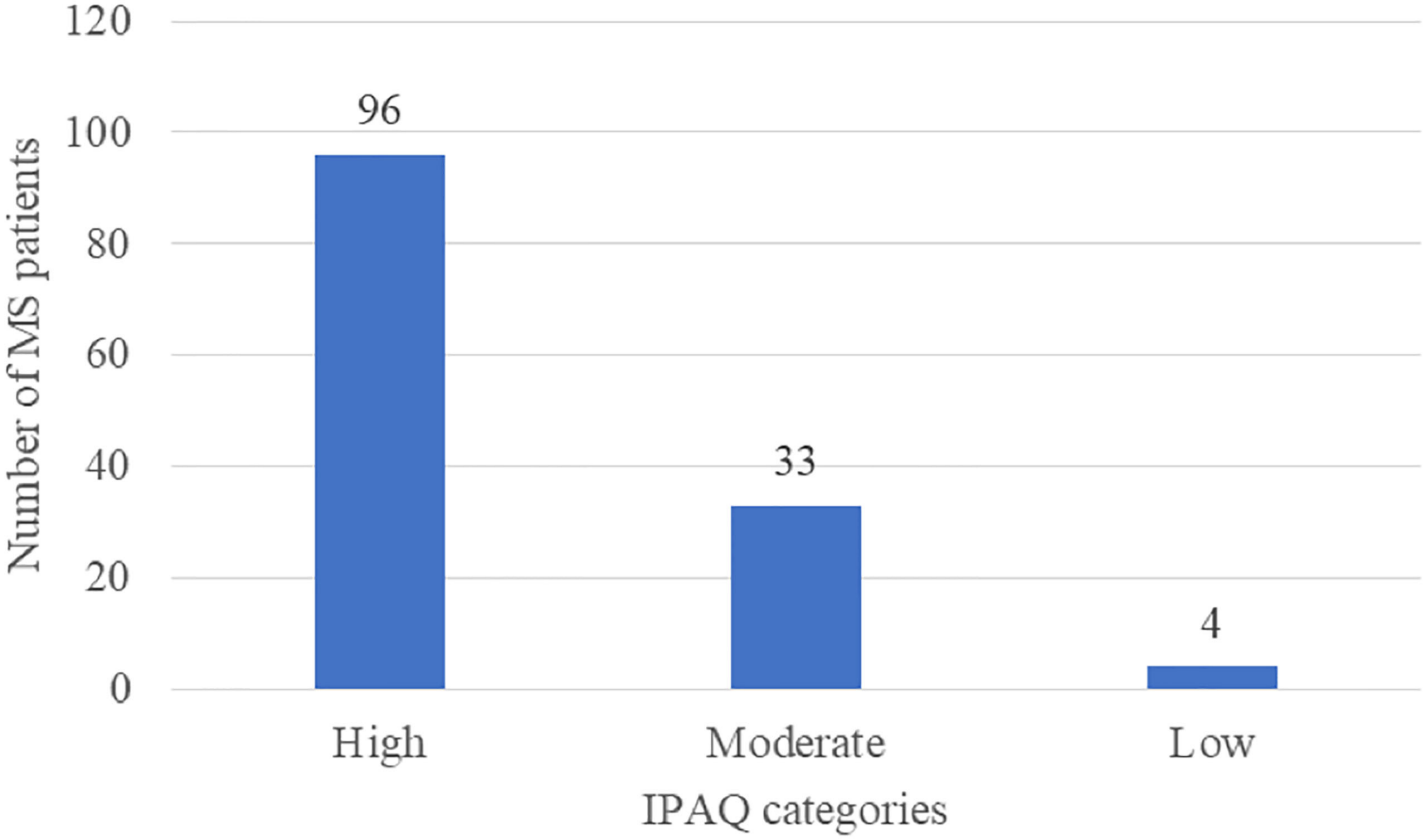
Levels of Physical Activities among pwMS.

The scores of IPAQ were also calculated to measure the prevalence of the three categories of physical activities as well as the total physical activities for patients. The median vigorous MET-minutes/week score was 960 (IQR = 480 – 2880), median moderate MET-minutes/week was 600 (IQR = 1200 – 240), and median walking MET-minutes/week was 594 (IQR = 1039 – 239). The median total physical activity MET-minutes/week score was 2838 (4802 – 1364). Physical activities from IPAQ were also categorized into high (i.e., total physical activity of at least 1500 MET-minutes/week), moderate (i.e., total physical activity of at least 600 MET-minutes/week), and low (i.e., patients who did not meet high and moderate levels) as indicated in **Figure 3**. It seems that the majority of the participants who performed any level of physical activities were engaged in a high level of physical activity (n = 94, 72.2%). While, 33 patients exhibited a moderate level of physical activity (24.8%), and a very small number of patients had indicated a low level of physical activity (n = 4, 3.0%). There were no statistically significant differences in median total physical activity MET-minutes/week by patients’ characteristics (p-value > 0.05). However, time since disease diagnosis was positively and significantly correlated with total physical activity MET-minutes/week (r = 0.203, p-value = 0.019) as shown in **Table 6**.

**Table 6:** Correlations Between Depression, Anxiety, EQ-VAS, FSS, and IPAQ.

	Depression	Anxiety	FSS	IPAQ	EQ-VAS
**Disease duration (in years)**	-0.071	-0.112*	-0.044	0.203*	0.063
**Depression**		0.655*	0.407*	- 0.087	- 0.479*
**Anxiety**			0.289*	- 0.069	- 0.497*
**FSS**				- 0.123	- 0.336*
**IPAQ**					0.064

*Significant at 5% level of significance.

### Correlations Between Depression, Anxiety, EQ-VAS, FSS, and IPAQ

The correlation between the measures used in this study are shown in **Table 6**. The results reveal that FSS and depression were positively and significantly correlated (r = 0.407, *P*-value < 0.001), which indicates that a higher FSS level is associated with a higher level of depression. Similarly, a positive and significant correlation between the FSS and anxiety levels was detected (r = 0.289, *P*-value < 0.001), which suggests that higher FSS scores are associated with higher scores of anxiety. Furthermore, the correlation between depression and anxiety was significant and positive indicating that a higher score of depression is associated with a higher score of anxiety (r = 0.655, *P*-value < 0.001). However, the total IPAQ score was not significantly correlated with depression (r = - 0.087, *P*-value = 0.319), anxiety (r = - 0.069, *P*-value = 0.433), or fatigue (r = - 0.123, *P*-value = 0.158). Moreover, a negative and significant correlation was found between EQ-VAS and depression (r = -0.479, *P*-value < 0.001), anxiety (r = - 0.497, *P*-value < 0.001), and FSS (r = - 0.336, *P*-value < 0.001) but was not significantly associated with IPAQ (r = 0.064, *P*-value = 0.467) and time since disease diagnosis (r = 0.063, *P*-value = 261).

## Discussion

This study shows that patients in our cohort report anxiety or depression as measured by the HADS instrument. Moreover, the average score of depression falls within the normal category. However, the average score of anxiety falls within the symptoms of a borderline case. This suggests that patients in our cohort were more likely to be anxious rather than depressed. The findings of this study are in accordance with previously published research (10, 13, 15, 16, 37) but are contradicted by some others (7, 11, 12, 14). In agreement with this study’s findings, for instance, a study conducted in the neighboring United Arab Emirates reported that depression and anxiety were present among 17% and 20% of patients with MS respectively (10). Nevertheless, a systematic review and meta-analysis found that the prevalence of depression was 30.5% among pwMS while the prevalence of anxiety was 22.1% among them, which is not congruent with the current study findings (7).

The results of this study also indicate that depression and anxiety scores did not correlate with gender, marital status, nationality, education, or work status. However, pwMS from central and western regions exhibited higher mean scores of depression and anxiety than those from the eastern region, which might be attributed to the highly urban and increased industrialized lifestyle in the eastern region compared to the western and central region. Patients in the youngest and oldest age groups in our study exhibited higher average anxiety scores than those aged 30 – 39 years but they reported similar depression levels. Furthermore, disease duration was negatively associated with anxiety but not with depression. Łabuz-Roszak et al. found that in pwMS, depression correlated significantly with age, professional status, and educational levels, while anxiety was significantly associated with age and professional status. Nonetheless, they showed that both depression and anxiety were not significantly associated with gender or disease duration (16). However, others have shown that anxiety was more common among women and in those with a history of depression but it did not appear to be associated with age, education, work status, marital status, disease duration, or living status (15). On the other hand, Alsaadi et al. showed that depression and anxiety were not significantly associated with age, gender, education, disease duration, expanded disability status Stage (EDSS), or marital status (10). In two previous studies from Norway, anxiety and depression were more common in pwMS than controls, however, similar to our study, they did not correlate with gender or disease duration (12, 14). A longitudinal study in Southern Tasmania indicated that females were more anxious and depressed than males at cohort entry but this effect was not statistically significant (11). Therefore, the findings of previous studies were not conclusive that could be due to the different study variables; including measurement tool and patient population.

In this study, the QoL was also assessed by the EQ-5D-3L instrument. Most participants exhibited a healthy state as measured by EQ-5D-3L VAS with a mean score of 80.43 (SD = 19.8), which is higher than that reported by Algahtani et al., who estimated it as 73.87 (SD = 23.41) among pwMS in King Abul-Aziz Medical City in Saudi Arabia (38). The findings of this study indicate that decreasing MS relapses is associated with better health outcomes, which is congruent with various previous research (15, 23, 39, 40). Gupta et al. indicated that the higher rates of MS severity were associated with the worst health outcomes (40).

The present study also shows that the prevalence of fatigue was 48.3% of pwMS, whereby it was more present in women, younger patients, married, Saudis, those living in the central region, university educated, and in patients with normal depression and anxiety. However, fatigue was not significantly associated with age, gender, marital status, nationality, region, education, work status, and disease duration but was significantly associated with anxiety and depression measured by HADS and QoL measured by EQ-VAS. The findings are, to some extent, consistent with other research (6, 12, 14, 16, 17, 23, 41). We find an almost similar prevalence of fatigue to that reported in other studies. For instance, Rzepka et al. detected fatigue in 42% of patients with MS (17). Runia et al. reported the presence of fatigue in 46.5% of patients with MS (41). However, in other research, the prevalence of fatigue is higher, ranged, on average, between 50 − 80% because in patients our cohort was mildly affected and mobile (6, 16, 42). In Poland, for instance, fatigue was prevalent in 61.5% of pwMS and was not significantly associated with age, gender, disease duration, and course, EDSS, education but it was significantly correlated with depression, anxiety, sleep disorder, and professional status (16). Lerdal et al. showed that fatigue was prevalent among 61.1% of pwMS and was negatively correlated with their education but positively correlated with age and disease duration, which contradicted our results (42). A recent study by Rzepka et al. did not find any significant differences in FSS by gender, age, marital status, place of residence, which is consistent with our findings. However, it did find significant differences in FSS by education, EDSS, disease duration, professional status, IPAQ, which contradicted our results (17). Fidao et al. showed that the fatigue prevalence ratio was higher among pwMS with high depression risk, severe disability, obesity, smokers, and unemployed but was lower among pwMS with university education and higher IPAQ (18).

Moreover, the present study also indicates that 96 out of 133 (72.2%) pwMS in Saudi Arabia were engaged with a high level of physical activity. The total IPAQ score was positively and significantly correlated with disease duration, but it was not significantly associated with depression, anxiety, FSS, and EQ-VAS, as well as did not significantly differ by age, gender, marital status, nationality, region, education, and work status. This could be due to the low number of respondents on the IPAQ, where only 133 out of 323 patients had answered that part of the questionnaire. A study in Poland indicated that the prevalence of high physical activity among pwMS was 45%, which is lower than our findings. Moreover, a significant correlation between IPAQ and FSS scores was detected, which contradicted our results (17). A study by Fidao et al. showed that the prevalence ratio fatigue among pwMS was significantly lower for patients engaged with high physical activity in comparison to those inactive and minimally active patients, which is not consistent with our findings (18). Marck et al. exhibited that the increased levels of physical activity measured by IPAQ have significantly improved the QoL (43). A recent study by Reguera-García et al. reported that 33.3% and 34.3% of pwMS have vigorous and moderate levels of physical activity during COVID-19 outbreak, respectively measured by IPAQ-short form (44).

The correlations between depression, anxiety, and fatigue were also assessed in our cohort of patients. The results of this study suggested moderate correlations between depression and anxiety as well as depression and fatigue but a weak correlation was found between anxiety and fatigue. Previous studies reported significant inter-correlation between depression, anxiety, and fatigue in terms of symptoms cluster approach (22, 45–51). For instance, a study by Motl and McAuley assessed the symptom cluster of fatigue, pain, and depression as a correlate of decreased QoL in the pwMS cohort. They showed that high QoL was associated with low levels of fatigue, pain, depression, and vice versa (45). Brown et al. conducted a longitudinal study in Australia and found that anxiety and fatigue were substantially predicted by depression, while later depression was considerably predicted by anxiety and fatigue. Other factors including combinations of unhealthy behaviors such as smoking, drug use, no exercise, or relaxation, and psychological factors such as low optimism, avoidance coping were significantly predicted psychological distress (i.e., depression and anxiety). Meanwhile, immunotherapy status was significantly associated with fatigue and state anxiety as well as fatigue was predicted by patients’ demographics and life-event stressors (46). Chalah et al. reported that there is a bidirectional relationship between fatigue and neuropsychological factors (i.e., anxiety, depression, and alexithymia) (47).

It is important to note that the MS-related literature has tried to illustrate the associations between these adverse outcomes from different perspectives and mechanisms that underlie them (22). From a psychological perspective, anxiety, depression, and fatigue had a bidirectional relationship that might be explained by patients’ cognitions, emotions, and behaviors (22, 46–48, 52). For instance, Schreiber et al. indicated that anxiety, depression, and somatic symptoms were considered as relevant mediators of fatigue (48). From a pathophysiological perspective, however, some studies pointed out that anxiety and depression in pwMS were associated with pathologies including the frontal lobes and/or their connections (22, 50, 53–59). The development of depressive symptoms was also attributed to temporal, parietal, and limbic abnormalities (56). As for anxiety, some studies showed that the development of anxiety symptoms was associated with damage in septo-fornical in this cohort (50). Concerning fatigue, previous research documented that it has been associated with neural substrates involving the cortico-thalamocortical loop as the basis for developing fatigue in pwMS. Such a loop revealed several cortical and subcortical areas, of which the frontoparietal regions and/or their connections are largely involved (22, 58, 59). On the other hand, from a therapeutic perspective, some studies indicated that anxiety, depression, and fatigue treatments and medications may not be feasible and suggested further treatment modalities that should be investigated (22, 60). Considering these mechanisms and the fact that some neural hubs are common for various symptoms, damage that occurred in these hubs would lead to a cluster of complaints and hence might explain the joint incidence of psychological distress and fatigue (22, 54). Therefore, further studies are needed to assess the underlying mechanisms of these symptoms.

The results reveal that about one-half of respondents were not engaged in physical activity. This could be resolved by educational programs for pwMS to improve their coping with the disease. Therefore, further research is needed to identify this problem. Furthermore, it would be useful to conduct longitudinal research with follow-up pwMS and better to include variables like coping strategies, resilience, sense of cohesion, and some more precise physical capacities rehabilitated by the disease. It is important to note that depression, anxiety, and fatigue will be probably higher if pwMS who have a motor disability were included.

The current study has some limitations. First, this study measures only self-reported assessments of fatigue, depression, anxiety, QoL, and physical activity among patients with MS, not actual psychiatric diagnoses. Nevertheless, the instruments exhibited robust psychometric characteristics that have been applied across a wide range of studies (14, 29, 33, 35, 61, 62). Furthermore, the current study assessed the cross-sectional prevalence of the pwMS cohort and lacks some important clinical variables including MS type, immunotherapy, disease-specific treatments or medications, and Expanded Disability Status Scale (EDSS) score as well as it did not control for potential confounders. Therefore, further studies should tackle these variables that might improve the results. Second, the questionnaire used does not specifically identify the type of physical activity which would inform decisions. Third, the current study was based on subjective assessment and self-reporting of fatigue and thus was not able to identify its clear causes whether it was simply due to MS complications such as sleep disorders, endocrine dysfunction, and mood disorders, or rather a primary MS fatigue (22). Finally, this study did not take into account the role of social support on coping strategies for MS patients, which might affect the quality of the results (21).

## Conclusion

The current study shows that depression, anxiety, and fatigue, are frequent among pwMS in Saudi Arabia. Moreover, fatigue is associated positively with anxiety and depression, negatively with EQ-VAS, but not with physical activity. The results of this study are important for the improvement of the clinical management of MS patients. Furthermore, potential clinicians should have more focus on anxiety and depression symptoms among people with MS disease to develop appropriate treatments for those patients. Finally, support programs should be made available for pwMS to ensure adequate coping with the disease.

## Data Availability Statement

The original contributions presented in the study are included in the article/supplementary material. Further inquiries can be directed to the corresponding author.

## Author Contributions

SAS and NA conceived of the presented idea. AA, TA and SAS planned and carried out the simulations. RA, HA and SAS developed the theory and performed the computations. NA contributed to the interpretation of the results. NA and SAJ verified the analytical methods. RA and SAS investigate and supervised the findings of this work. SAS carried out the experiment. SAS and NA wrote the manuscript. All authors provided critical feedback and helped shape the research, analysis and manuscript. All authors discussed the results and contributed to the final manuscript.

## Funding

King Fahad Medical City (Grand number 019-058).

## Conflict of Interest

The authors declare that the research was conducted in the absence of any commercial or financial relationships that could be construed as a potential conflict of interest.

## Publisher’s Note

All claims expressed in this article are solely those of the authors and do not necessarily represent those of their affiliated organizations, or those of the publisher, the editors and the reviewers. Any product that may be evaluated in this article, or claim that may be made by its manufacturer, is not guaranteed or endorsed by the publisher.

## Abbreviations

ARFA MS, Saudi MS Association that is approved from Saudi Ministry of Human Resources and social development; EQ-5D-3L, EuroQOL five-dimension questionnaire; EQ-VAS, EQ Visual Analogue Scale; FSS, Fatigue Severity Scale; HADS, Hospital Anxiety and Depression Scale; IPAQ, International Physical Activity Questionnaire; IQR, Interquartile range; KFMC, King Fahd Medical City; KSA, Kingdom of Saudi Arabia; KSUMC, King Saud University Medical City; MS, Multiple Sclerosis; NMSR, National MS Registry; PwMS, Patient with multiple sclerosis; QoL, Quality of life; SD, Standard Deviation; SPSS, Statistical Package for Social Sciences; VAS, Visual analogue scale.
